# Sex-specific up-regulation of lncRNAs in peripheral blood of patients with schizophrenia

**DOI:** 10.1038/s41598-019-49265-z

**Published:** 2019-09-04

**Authors:** Hamid Fallah, Iman Azari, Seyedeh Morvarid Neishabouri, Vahid Kholghi Oskooei, Mohammad Taheri, Soudeh Ghafouri-Fard

**Affiliations:** 1 Department of Medical Genetics, Shahid Beheshti University of Medical Sciences, Tehran, Iran; 2 Department of Psychiatry, Shahid Beheshti University of Medical Sciences, Tehran, Iran; 3 Department of Laboratory Sciences, School of Paramedical Sciences, Torbat Heydariyeh University of Medical Sciences, Torbat Heydariyeh, Iran; 4 Neuroscience Research Center, Torbat Heydariyeh University of Medical Sciences, Torbat Heydariyeh, Iran; 5 Urogenital Stem Cell Research Center, Shahid Beheshti University of Medical Sciences, Tehran, Iran

**Keywords:** Molecular neuroscience, Gene expression

## Abstract

Schizophrenia as a common disabling psychiatric disorder has been associated with dysregulation of several genes and pathways among them are those being regulated by long non-coding RNAs (lncRNAs). Based on the acknowledged roles of lncRNAs in neurodevelopment, in the current study, we assessed expression of six lncRNAs namely *HOXA-AS*2, *Linc-ROR*, *MALAT1*, *MEG3*, *SPRY4-IT1* and *UCA1* in peripheral blood of 60 patients with schizophrenia and 60 healthy subjects. *HOXA-AS2*, *Linc-ROR*, *MEG3*, *SPRY4-IT1* and *UCA1* levels were significantly higher in total patients compared with total controls. However, when evaluating expression of genes in sex-based subgroups, the differences in the expression of these lncRNAs were significant only among females. Assessment of partial correlation between expression of lncRNAs and age of study participants after controlling the effect of sex, revealed significant correlations for *HOXA-AS2*, *MALAT1* and *UCA1* in both patients and controls. Besides, expressions of *Linc-ROR* and *SPRY4-IT1* were correlated with age only in patients. Significant pairwise correlations were recognized between expression levels of lncRNAs in both patients with schizophrenia and controls. Based on the area under curve (AUC) values, *SPRY4-IT1* had the best performance in differentiation of female patients with schizophrenia from female controls (AUC = 0.85, P < 0.0001). Combination of *Linc-ROR*, *MEG3*, *SPRY4-IT1* and *UCA1* expression levels could differentiate female patients with 95.2% sensitivity, 76.9% specificity and diagnostic power of 0.88 (P < 0.0001). The current study suggests the presence of a sex-based dysregulation of lncRNAs in patients with schizophrenia and their possible application as diagnostic biomarkers.

## Introduction

Schizophrenia is a devastating psychiatric disorder which affects approximately 1% of individuals throughout their lifespan. This disorder is characterized by the existence of an arrangement of symptoms including positive symptoms (hallucinations, delusions, abnormal concentrating and movement disorder), negative symptoms (apathy, lack of pleasure, avolition, and flattening), and cognitive symptoms (defects in administrative function and attention)^1^. Although the main cause of schizophrenia has not been recognized yet, there is a bulk evidences indicating the role of gene expression dysregulation in the pathogenesis of this disorder^2–4^. Long non-coding RNAs (lncRNAs) are the major regulators of gene expression which execute this role via binding to histone-modifying proteins, transcription factors and RNA polymerase II^5^. Numerous expression profiling studies have demonstrated aberrant expression of lncRNAs in the peripheral blood and the brain tissues of patients with schizophrenia^6,7^. However, the role of some other lncRNAs in the pathogenesis of this psychiatric disorder has not been explored. In the current study, we have used a literature-based method to identify lncRNAs with putative but indirect or unappreciated roles in schizophrenia. We selected six lncRNAs namely *HOXA-AS2*, *Linc-ROR*, *MEG3*, *SPRY4-IT1*, *UCA1* and *MALAT1* to assess their expression in peripheral blood of patients with schizophrenia and healthy subjects. Any of the lncRNAs selected for this investigation affects one plausible aspect of schizophrenia pathogenesis. *HOXA transcript antisense RNA 2* (*HOXA-AS2*) is transcribed from *HOXA* cluster between and antisense to the human *HOXA3* and *HOXA4* genes^8^. Considering the role of *HOXA* genes in the process of neurodevelopment^9^ and the role of abnormal brain development in the pathogenesis of schizophrenia^10^, *HOXA-AS2* might be involved in this psychiatric disorder. *The long intergenic non-protein coding RNA, regulator of reprogramming* (*Linc-ROR*) controls the reprogramming of pluripotent stem cells^11^ and is regarded as an inhibitor of p53 tumor suppressor^12^, a gene which has been believed to contribute in schizophrenia for a long time^13^. The *maternally expressed gene 3* (*MEG3*) regulates expression of AMPA glutamate receptor in primary cortical neurons^14^. Based on the reported abnormalities in the glutaminergic system in schizophrenia^15^, *MEG3* is another putative lncRNA in the pathogenesis of schizophrenia. The *SPRY4 intronic transcript 1* (*SPRY4-IT1*) regulates levels of lipin 2, a protein that facilitates conversion of phosphatidate to diacylglyceroland. *SPRY4-IT1* possibly participates in lipid biosynthesis as its silencing leads to lipotoxicity^16^. Considering the observed associations between altered lipid profile and occurrence of schizophrenia^17^, *SPRY4-IT1* might be involved in this disorder. The *urothelial cancer associated 1* (*UCA1*) has an established role in neurons as it suppresses apoptosis of hippocampal neurons via miR-495/Nrf2-ARE pathway^18^. Moreover, this lncRNA contributes in inhibition of hypoxia injury after cerebral ischemia^19^. As schizophrenia is known as “an adult vascular-ischemic disorder”^20^, *UCA1* might affect the course of this disorder. Finally, *Metastasis Associated Lung Adenocarcinoma Transcript 1* (*MALAT1*) is abundantly expressed in neurons and is enriched in nuclear speckles in a transcription-dependent manner., *MALAT1* participates in the regulation of synaptogenesis-related genes *in vitro*^21^. Besides, its silencing has decreased synaptic density in cultured hippocampal neurons^21^, a finding that potentiates this lncRNA as a modulating agent in the course of schizophrenia based on the reported decrease in synaptophysin in hippocampus and frontal cortical areas of patients with schizophrenia^22^. Consequently, dysregulation of the selected lncRNAs might be involved in the schizophrenia pathogenesis or applied as disease biomarkers.

## Material and Methods

### Study participants

The current study was performed in 60 patients with schizophrenia (39 male patients and 21 female patients, mean age ± standard deviation: 49.63 ± 9.64) and 60 healthy subjects (47 male patients and 13 female patients, mean age ± standard deviation: 49.48 ± 11.94). Patients were referred to psychiatry departments of Shahid Beheshti and Hamadan Universities of Medical Sciences. Patients were diagnosed according to the fifth edition of Diagnostic and Statistical Manual of Mental Disorders (DSM-V)^1^. Patients with schizophrenia were taking standard dose of Clozapine™ (301 mg/day to 600 mg/day). Inclusion criteria were compliance with the diagnostic criteria and patients’ willingness for participation in the study. Exclusion criteria were substance abuse, cigarette smoking or use of other antipsychotic drugs. Persons enlisted as controls were assessed through a structured psychiatric interview (Mini-International Neuropsychiatric Interview^23^), for excluding the presence of psychiatric disorders. Exclusion criteria were the presence of malignancy, recent or continuous infectious disorder, autoimmune conditions, nerve muscle coupling disorders and pregnancy. The study protocol was approved by Ethical Committee of Shahid Beheshti University of Medical Sciences and all methods were performed in accordance with the relevant guidelines and regulations. Informed written consent forms were signed by all study participants.

### Expression study

Total RNA was isolated from venous blood of enrolled individuals using Hybrid-RTM blood RNA extraction Kit (GeneAll Biotechnology Co. Ltd., Seoul, South Korea). The quality and quantity of RNA was appraised using Nanodrop equipment (Thermo Scientific, MA, USA). Subsequently, cDNA was produced using FIREScript RT cDNA Synthesis Kit (Solis BioDyne, Estonia). Relative expressions of lncRNAs were assessed in patients with schizophrenia and controls using RealQ Plus Master Mix Green (AMPLICON, Denmark) in the rotor gene 6000 Real-Time PCR System (Corbett, Australia). *B2M* gene was used as the normalizer. The sequences of primers and amplicon lengths are shown in Table 1.

**Table 1:** Sequences of primers used in the study.

Primer Name	Sequence	Primer Length	PCR Product Length
MEG3-F	TGGCATAGAGGAGGTGAT	18	111
MEG3-R	GGAGTGCTGTTGGAGAATA	19	
SPRY4-IT1-F	AGCCACATAAATTCAGCAGA	20	115
SPRY4-IT1-R	GATGTAGGATTCCTTTCA	18	
HOXA-AS2-F	CCCGTAGGAAGAACCGATGA	20	70
HOXA-AS2-R	TTTAGGCCTTCGCAGACAGC	20	
Linc-ROR-F	TATAATGAGATACCACCTTA	20	170
Linc-ROR-R	AGGAACTGTCATACCGTTTC	20	
UCA1-F	CTTAGGCTGGCAACCATCAGATCC	24	129
UCA1-R	GTGTTGTCCTGCATGCTGGTCTG	23	
MALAT1-F	GACGGAGGTTGAGATGAAGC	20	84
MALAT1-R	ATTCGGGGCTCTGTAGTCCT	20	
B2M-F	AGATGAGTATGCCTGCCGTG	20	105
B2M-R	GCGGCATCTTCAAACCTCCA	20	

### Statistical methods

Expressions of lncRNAs in each sample were calculated using the Efficiency ^^Ct^ normalizer gene-Efficiency ^^CT^ target gene method. The Statistical Package for the Social Sciences (SPSS) v.18.0 (SPSS Inc., Chicago, IL) was used for statistical assessments. The correlations between transcript levels of lncRNAs were evaluated using regression model and Bonferroni correction for multiple comparisons. The correlation between expression levels and age of study participants was described by R and P values. Mean values of gene expression were compared between education-based subgroups of patients and controls using one-way ANOVA and Tukey post hoc tests. For all statistical tests, the level of significance was set at P < 0.05. The receiver operating characteristic (ROC) curves were depicted to appraise the diagnostic power of expression levels of lncRNAs.

## Results

### General data of study participants

The available data of study participants are summarized in Table 2.

**Table 2:** General data of study participants.

Study groups	Parameters		Values
Patients	Sex (number)	Male	39
		Female	21
	Age (Years, mean ± SD (range))	Male	51.25 ± 10.38 (32–79)
		Female	46.61 ± 7.37 (31–61)
	Age at onset (Years, mean ± SD (range))	Male	34.94 ± 1.86 (29–39)
		Female	35.09 ± 2.47 (29–40)
	Duration (Years, mean ± SD (range))	Male	16.73 ± 9.55 (1–46)
		Female	11.52 ± 6.08 (1–22)
	Education (%)	Preschool	30%
		School	48.3%
		University	21.7%
Controls	Sex (number)	Male	47
		Female	13
	Age (Years, mean ± SD (range))	Male	50 ± 12.75 (25–77)
		Female	49.63 ± 8.58 (34–61)
	Education (%)	Preschool	11.6%
		School	26.7%
		University	61.7%

**Figure 1:**
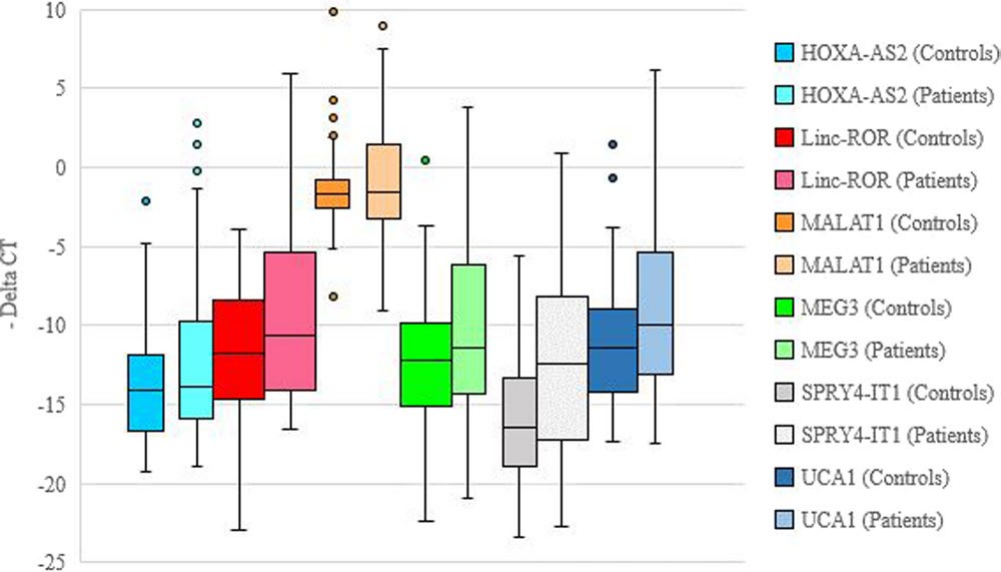
Relative expression of lncRNAs in patients with schizophrenia and controls as described by –delta Ct values (Ct Housekeeping gene- Ct Target gene).

When comparing expression of lncRNAs in total patients and total controls, *HOXA-AS2*, *Linc-ROR*, *MEG3*, *SPRY4-IT1* and *UCA1* were significantly over-expressed in patients. However, when evaluating expression of genes in sex-based subgroups, the differences in the expression of these lncRNAs were significant only among females. Table 3 shows the expression ratios and P values of comparison of genes expressions between groups. Figure 1 depicts the –delta Ct values of lncRNAs in patients and controls.

**Table 3:** The results of expression study of lncRNAs in peripheral blood of patients with schizophrenia compared with controls (The statistical power values for comparison between female cases and female controls as computed by Post Hoc analysis are 86% (*HOXA-AS2*), 99.6% (*Linc-ROR*), 60% (*MALAT1*), 97.4% (*MEG3*), 98.7% (*SPRY4-IT1*) and 98.6% (*UCA1*) respectively).

		Total patients vs. controls (60 vs. 60)	Male patients vs. male controls (39 vs. 47)	Female patients vs. female controls (21 vs. 13)
*HOXA-AS2*	Expression ratio	2.88	1.28	12.84
	P-value	0.04	0.7	0.03
*Linc-ROR*	Expression ratio	4.44	1.4	53.07
	P-value	0.008	0.55	0.005
*MALAT1*	Expression ratio	1.42	0.82	3.64
	P-value	0.34	0.53	0.12
*MEG3*	Expression ratio	3.17	1.27	18.93
	P-value	0.02	0.67	0.01
*SPRY4-IT1*	Expression ratio	6.24	1.87	87.14
	P-value	0.001	0.3	<0.001
*UCA1*	Expression ratio	4.16	1.63	31.23
	P-value	0.006	0.34	0.009

### Correlation between expression of lncRNAs and age of study participants

Assessment of partial correlation between expression of lncRNAs and age of study participants after controlling the effect of sex, revealed significant correlations for *HOXA-AS2*, *MALAT1* and *UCA1* in both patients and controls. Besides, expressions of *Linc-ROR* and *SPRY4-IT1* were correlated with age only in patients. Expressions of *HOXA-AS2*, *MEG3* and *UCA1* were correlated with age at disease onset in patients with schizophrenia. Moreover, expressions of *MALAT1* and *UCA1* were correlated with disease duration in these patients. Table 4 shows the results of partial correlation between expression of lncRNAs and age.

**Table 4:** The results of partial correlation between expression of lncRNAs and age (controlled for sex).

Study groups	Parameters	*HOXA-AS1*		*Linc-ROR*		*MALAT1*		*MEG3*		*SPRY4-IT1*		*UCA1*	
		R	P value	R	P value	R	P value	R	P value	R	P value	R	P value
Patients	Age	0.27	0.02	0.25	0.03	0.27	0.02	0.2	0.06	0.22	0.04	0.4	0.001
	Age at onset	0.28	0.01	0.16	0.11	0.18	0.08	0.23	0.04	0.17	0.1	0.32	0.007
	Disease duration	0.2	0.07	0.02	0.07	0.21	0.03	0.12	0.18	0.16	0.11	0.33	0.006
Controls	Age	0.37	0.002	0.07	0.3	0.32	0.007	0.16	0.1	0.15	0.12	0.25	0.03

### Correlation between expression levels of lncRNAs

Significant pairwise correlations were recognized between expression levels of lncRNAs in both patients with schizophrenia and controls (Table 5).

**Table 5:** Correlations between expressions of lncRNAs in study groups (R^2^ values are presented; after correction for multiple comparisons (Bonferroni correction), P value less than 0.0016 was accepted as significant (marked by *)).

		*UCA1*	*SPRY4-IT1*	*MEG3*	*MALAT1*	*Linc-ROR*
HOXA-AS2	Controls	0.4*	0.21*	0.31*	0.26*	0.1
	Patients	0.5*	0.5*	0.62*	0. 5*	0.68*
Linc-ROR	Controls	0.22*	0.14	0.19*	0.12	
	Patients	0.31*	0.57*	0.6*	0.37*	
MALAT1	Controls	0.22*	0.2*	0.18		
	Patients	0.31*	0.26*	0.32*		
MEG3	Controls	0.24*	0.21*			
	Patients	0.36*	0.48*			
SPRY4-IT1	Controls	0.24*				
	Patients	0.23*				

### Association between expression level of genes and education level

We compared mean values of gene expression between education-based subgroups of patients and controls using one-way ANOVA test. The results of Tukey post hoc test showed significant difference in *SPRY4-IT1* expression between preschool and school groups in normal individuals. Expressions of other genes were not different between education-based subgroups of either patients or controls (Table 6).

**Table 6:** Mean values (±standard deviation) of gene expressions in different subgroups of patients and controls. Study participants are stratified based on their higher level of education.

Genes	Controls				Patients			
	Preschool	School	University	P value	Preschool	School	University	P value
*HOXA-AS1*	15.22 ± 3.09	13.17 ± 4.33	13.84 ± 3.5	0.47	12.44 ± 5.8	11.9 ± 6.3	11.9 ± 5.37	0.95
*Linc-ROR*	13.01 ± 5.63	11.93 ± 4.41	11.58 ± 4.35	0.74	8.86 ± 6.79	9.25 ± 5.82	9.96 ± 5.63	0.88
*MALAT1*	3.38 ± 2.83	1.32 ± 2.11	1.03 ± 2.88	0.11	−0.26 ± 3.6	1.32 ± 4.25	1.26 ± 4.37	0.4
*MEG3*	12.8 ± 3.51	11.4 ± 5.32	12.44 ± 3.97	0.67	10.64 ± 7.18	9.98 ± 5.07	10.19 ± 5.5	0.93
*SPRY4-IT1*	18.82 ± 2.46	14.15 ± 4.55	15.77 ± 4.02	0.04	13.04 ± 5.34	12.33 ± 6.09	12.36 ± 5.07	0.9
*UCA1*	12.44 ± 3.12	10.36 ± 3.69	11.41 ± 4.32	0.49	10.35 ± 4.3	8.18 ± 5.8	8.05 ± 6.05	0.36

### ROC curve analysis

**Figure 2:**
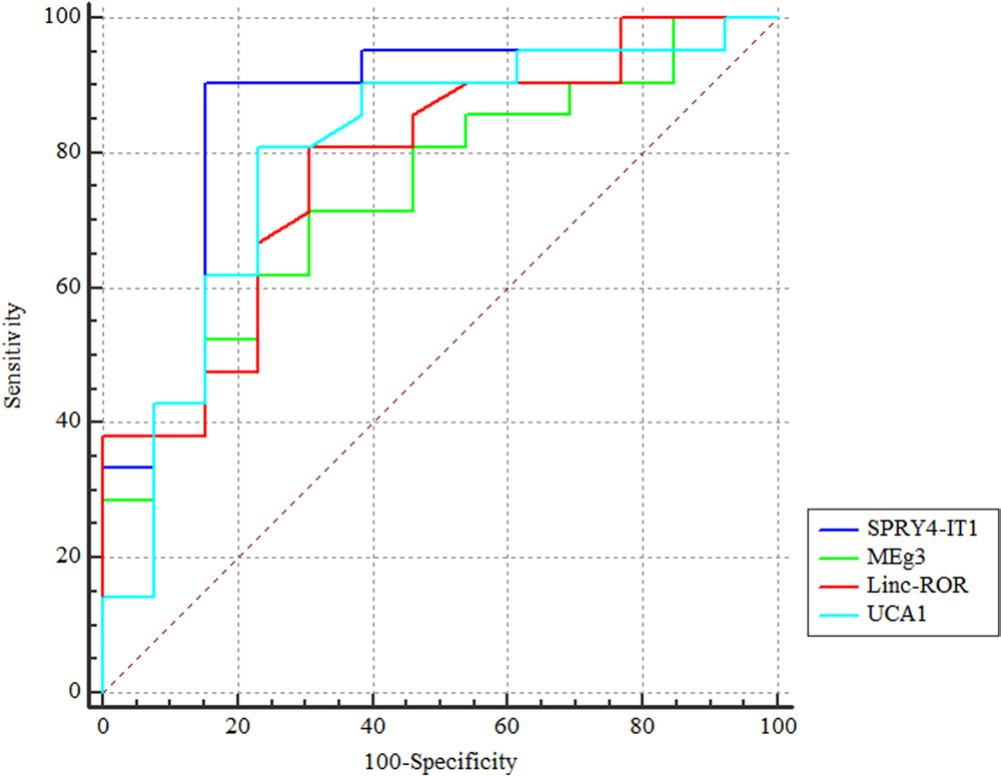
ROC curves of lncRNAs transcript levels in female subjects.

Based on the results of sex-based analysis and similar expression of genes between male patients and male controls, ROC curves were depicted only for female subjects (Fig. 2).

Table 7 shows the detailed information of ROC curve analysis. Based on the area under curve (AUC) values, *SPRY4-IT1* had the best performance in differentiation of female patients from female controls (AUC = 0.85, P < 0.0001). Combination of *Linc-ROR*, *MEG3*, *SPRY4-IT1* and *UCA1* expression levels could differentiate female patients with 95.2% sensitivity, 76.9% specificity and diagnostic power of 0.88 (P < 0.0001).

**Table 7:** The results of ROC curve analysis in female subjects.

	Estimate criterion	AUC	J	Sensitivity	Specificity	P-value
*Linc-ROR*	≤11.2	0.78	0.5	81	69.2	0.0006
*MEG3*	≤11.5	0.74	0.41	71.4	69.2	0.005
*SPRY4-IT1*	≤12.9	0.85	0.75	90.5	84.6	<0.0001
*UCA1*	≤12.1	0.8	0.58	81	76.9	0.0005
Combination of all genes	>0.46	0.88	0.73	95.2	77	<0.0001

## Discussion

In the current investigation, we reported significant up-regulation of *HOXA-AS2*, *Linc-ROR*, *MEG3*, *SPRY4-IT1* and *UCA1* in female patients compared with female normal subjects. However, expression levels of none of lncRNAs were different between male patients and male controls. The sex-based alterations have been noted previously in age of onset, course, and treatment response in patients with schizophrenia^24^. In addition, differences in neurodevelopmental processes and psychosocial parameters^24^ and increase in brain gene expression divergence in males have been associated with risk of schizophrenia^25^. The observed sex-based differences in the expression of lncRNAs in the current study might reflect the presences of hormone-response elements in the mentioned lncRNAs. Such elements have been noted in some lncRNAs such as *GAS5*^26^. Moreover, several lncRNAs are involved in germ cell specification, sex determination and sex hormone responses^27^. Two previously appreciated mechanisms for the observed sex-based differences in human diseases are gonadal sex hormones or the sex chromosomes^28^. The current study and similar studies in neuropsychiatric disorders^29^ provide preliminary evidences for contribution of lncRNAs in this process. However, further studies are required to appraise whether this effect is exerted through interaction with the previously appraised mechanisms or is performed independently.

Although males are expected to be affected with earlier onset and more severe disease course^30^, our findings revealed similar expression of lncRNAs between male patients and male controls. This finding possibly rules out the participation of these lncRNAs in the pathogenesis of schizophrenia in male subjects. Moreover, this observation is consistent with the results of our recent study where we reported significant associations between expression levels of *GAS5*, *NEAT1* and *OIP5-AS1* lncRNAs and schizophrenia in female subjects but not in male individuals^31^. The sharp sex-based contrasts in expression signatures of lncRNAs might imply the sex-specific roles for these lncRNAs. A recent study has revealed a sex-biased lncRNA signature in placenta^32^. Moreover, co-expression assessments have demonstrated several lncRNAs correlation with sex differences in mouse germline stem cells^33^. The importance of the observed sex-based differences in lncRNA patter in patients with schizophrenia is more highlighted when considering the extensive sex differences in gene signature in the adult human brain. Moreover, several genes with sex-biased expression are associated with diseases and possibly have functional significances. Taken together, such sex-biased expression implies the presence of sex-biased gene regulatory mechanisms^34^.

*HOXA-AS2* has been shown to interact with the enhancer of zeste homolog 2 (EZH2). EZH2 has crucial roles in the epigenetic silencing of cyclooxygenase-2^35^, an enzyme whose over-expression has been noted in schizophrenia as a result of immune response dysregulation^36^. Moreover, analysis of RNA-seq data has shown a significant elevation in *EZH2* levels in the anterior cingulate cortex of patients with schizophrenia compared to normal individuals. Based on these results, Billingsley *et al*. have suggested a role for EZH2 in schizophrenia. Such role might be exerted either through interference with normal brain development or through abnormal reactivation of expression in the CNS in the adulthood^37^. Future studies are necessary to find the interactions between *HOXA-AS2*, EZH2 and cyclooxygenase-2 in the context of schizophrenia.

*Linc-ROR* has been shown to act as a sponge for miR-138 and miR-145^38^. miR-138 has a regulatory role in dendritic spine morphogenesis^39^, a process that is dysregulated in schizophrenia^40^. miR-145 has a functional role in the brain tissue as its over-expression alleviates astrocyte damage in cerebral ischemic stroke^41^. This miRNA targets Aquaporin 4^41^, a gene whose polymorphisms are associated with negative symptoms of schizophrenia^42^. So, it is plausible that *Linc-ROR* participates in the pathogenesis of schizophrenia through modulation of miR-145 and subsequent alterations in Aquaporin 4. In addition, this lncRNA might affect schizophrenia through alterations in dendritic spine morphogenesis.

Elevated levels of *MEG3* have been reported in in the nucleus accumbens of heroin abusers^43^. Heroine influences dopaminergic, glutamatergic and GABAergic routes that participate in the pathogenesis of schizophrenia^44^. Consequently, it is possible that *MEG3* also affects the mentioned transmitters and participates in the evolution of schizophrenia.

Over-expression of *SPRY4-IT1* has been shown to induce EZH2^45^, a transcription factor that is over-expressed in the anterior cingulate cortex of patients with schizophrenia compared to controls and has been suggested to participate in the schizophrenia either via interference with developmental processes or through abnormal reactivation of gene expression in the adult brain^37^. This lncRNA might also contribute in schizophrenia through alteration of lipid profiles. Moreover, it is worth mentioning that both *SPRY4-IT1* and *HOXA-AS2* are partners of EZH2. So, dysregulation of these lncRNAs might have synergic effects on the aberrant expression of EZH2 in patients with schizophrenia.

*UCA1* is an lncRNA with regulatory roles on the Wnt/β catenin pathway^46^, a pathway that is highly dysregulated in peripheral blood of patients with schizophrenia^47^. Consequently, the observed over-expression of this lncRNA might participate in the pathogenesis of schizophrenia through alterations in Wnt/β catenin pathway. However, such expression pattern is not consistent with the proposed role for this lncRNA in protection against hypoxia^19^.

We also demonstrated significant correlations between age and expression of the lncRNAs *HOXA-AS2*, *MALAT1* and *UCA1* in both patients and controls. Besides, expressions of *Linc-ROR* and *SPRY4-IT1* were correlated with age only in patients. Such correlations might imply the role of age in determination of expression levels of these lncRNAs. This speculation is consistent with the results of a recent study which demonstrated an age-dependent diurnal expression of lncRNAs which concurs with age-related alterations in facultative heterochromatin^48^. We also reported correlations between expressions of *HOXA-AS2*, *MEG3* and *UCA1* and age at disease onset in patients with schizophrenia. Moreover, expressions of *MALAT1* and *UCA1* were correlated with disease duration in these patients. Such correlations might reflect the effects of disease course or antipsychotic treatments on genes expression. Alternatively, they might merely show the age-related mechanisms. Decisive results can be only obtained from larger-scale cohorts of patients with different age ranges and disease duration values.

We also demonstrated several pairwise correlations between expression of lncRNAs in both normal individuals and patients with schizophrenia which suggest their regulation by a similar possibly epigenetic mechanism or their involvement in similar cellular processes. Clues for the second possibility have been obtained for *SPRY4-IT1* and *HOXA-AS2* as both are EZH2 partners.

Finally, we reported the suitability of a panel of lncRNAs for discrimination of female patients with schizophrenia from normal female individuals. If these results are verified in larger sample sizes, the suggested panel might be used as a diagnostic panel for schizophrenia.

Taken together, our results imply contribution of certain lncRNAs in the pathogenesis of schizophrenia in female subjects and suggest them as elements of a diagnostic panel. However, our study has some limitations including lack of body mass index (BMI) and the Positive and Negative Syndrome Scale (PANSS) score of study participants and unavailability of drug-naïve patients. Although the effect of Clozapine on gene expression cannot be ruled out without analyses of expression profile of drug-naïve patients, a previous study has indicated that Clozapine monotherapy might induce only minor alterations in gene expression^49^. By excluding patients who used other antipsychotic drugs, we have minimized the confounding factors.
